# Spatial Re-Establishment Dynamics of Local Populations of Vectors of Chagas Disease

**DOI:** 10.1371/journal.pntd.0000490

**Published:** 2009-07-28

**Authors:** Heinrich zu Dohna, María C. Cecere, Ricardo E. Gürtler, Uriel Kitron, Joel E. Cohen

**Affiliations:** 1 Center for Animal Disease Modelling, Department of Veterinary Medicine, University of California Davis, Davis, California, United States of America; 2 Laboratory of Eco-Epidemiology, Department of Ecology, Genetics and Evolution, University of Buenos Aires, Ciudad Universitaria, Buenos Aires, Argentina; 3 Department of Environmental Studies, Emory University, Atlanta, Georgia, United States of America; 4 Laboratory of Populations, Rockefeller University and Columbia University, New York, New York, United States of America; National Institute of Allergy and Infectious Diseases, United States of America

## Abstract

**Background:**

Prevention of Chagas disease depends mainly on control of the insect vectors that transmit infection. Unfortunately, the vectors have been resurgent in some areas. It is important to understand the dynamics of reinfestation where it occurs. Here we show how continuous- and discrete-time models fitted to patch-level infestation states can elucidate different aspects of re-establishment. *Triatoma infestans*, the main vector of Chagas disease, reinfested sites in three villages in northwest Argentina after community-wide insecticide spraying in October 1992.

**Methodology/Principal Findings:**

Different methods of estimating the probabilities of bug establishment on each site were compared. The results confirmed previous results showing a 6-month time lag between detection of a new infestation and dispersal events. The analysis showed that more new bug populations become established from May to November than from November to May. This seasonal increase in bug establishment coincides with a seasonal increase in dispersal distance. In the fitted models, the probability of new bug establishment increases with increasing time since last detected infestation.

**Conclusions/Significance:**

These effects of season and previous infestation on bug establishment challenge our current understanding of *T. infestans* ecology and highlight important gaps in knowledge. Experiments necessary to close these gaps are discussed.

## Author Summary

Chagas disease is transmitted by blood-sucking bugs (vectors) and presents a severe public health threat in the Americas. Worldwide there are approximately 10 million people infected with Chagas disease, a disease for which there is currently no effective cure. Vector suppression is the main strategy to control the spread of this disease. Unfortunately, the vectors have been resurgent in some areas. It is important to understand the dynamics of reinfestation where it occurs. Here we show how different models fitted to patch-level bug infestation data can elucidate different aspects of re-establishment dynamics. Our results demonstrated a 6-month time lag between detection of a new infestation and dispersal events, seasonality in dispersal rates and effects of previous vector infestation on subsequent vector establishment rates. In addition we provide estimates of dispersal distances and the effect of insecticide spraying on rates of vector re-establishment. While some of our results confirm previous findings, the effects of season and previous infestation on bug establishment challenge our current understanding of *T. infestans* ecology and highlight important gaps in our knowledge of *T. infestans* dispersal.

## Introduction

*Trypanosoma cruzi* is the causative agent of Chagas disease in the Americas. Of approximately 10 million persons infected, 10–40% develop a clinically overt disease, affecting heart, digestive or neurological functions. *Trypanosoma cruzi* is transmitted widely in South America by *Triatoma infestans* (Klug), a blood-sucking reduviid bug. Interrupting the transmission cycle by screening blood donors and suppressing the vector is currently the major strategy for controlling Chagas disease [1].

Since *Triatoma infestans* occurs mainly in poor rural areas of South America where resources for vector control are limited, it is important to increase the efficiency of vector control. It is currently unknown what spatial and temporal pattern of repeated insecticide application maximizes its efficiency. Developing an optimal spraying strategy requires a detailed knowledge of the spatio-temporal scale of vector dynamics as well as the effects of insecticide spraying in the field. This knowledge cannot be gained in laboratory studies but instead requires analyzing field data that cover the spatial range of *T. infestans* dispersal and the temporal range of *T. infestans* population recovery.

Such data on *T. infestans* populations have been accumulated as part of a larger research endeavour, which started in the 1990s, on the reinfestation dynamics of *T. infestans* in northwest Argentina [2],[3],[4],[5]. Within our study area (northwest Argentina) the population of *T. infestans* was structured as a metapopulation (for definition see [6]). A suitable framework for analyzing these data is therefore metapopulation theory. One goal of metapopulation theory is to predict under which conditions a network of interacting local populations goes extinct [7],[8],[9]. The extinction threshold is governed by how the rates of extinction and establishment of local populations depend on the presence and absence of neighboring populations. The parameters driving extinction rates and establishment rates of local populations can be estimated either from a single snapshot of patch occupancy [10], or from data sampled at different times (longitudinal data) if the patch occupancy changes over time [11].

The data analyzed here are longitudinal abundance data of an expanding population. A previous analysis of the same data estimated rate parameters of a non-spatial metapopulation model and found a pronounced seasonality in bug establishment (Figure 3 in [12]). This seasonality is at odds with experimental studies of flight initiation [13] and dispersal flight [14] of *T. infestans* indicating that a more detailed analysis of the bug abundance data is necessary.

This study expands our previous analysis by considering spatial locations of sites explicitly. We address three aspects relevant for population control of *T. infestans* that were not addressed previously: (i) the establishment rate of new local populations as a function of distance from existing local populations, (ii) possible mechanisms for the observed seasonality in bug establishment, and (iii) the effect of insecticide spraying and previous infestation on establishment rates. Our data provided two challenges that have rarely been addressed previously for metapopulation data: the possibility of false negatives in patch occupancy data and the need to interpolate dispersal processes between surveys to estimate effects of insecticide spraying between surveys. We addressed these challenges by fitting several competing models to the data. The present study demonstrates how knowledge about a complex metapopulation system can be gained by fitting a range of competing models.

## Methods

**Figure 1:**
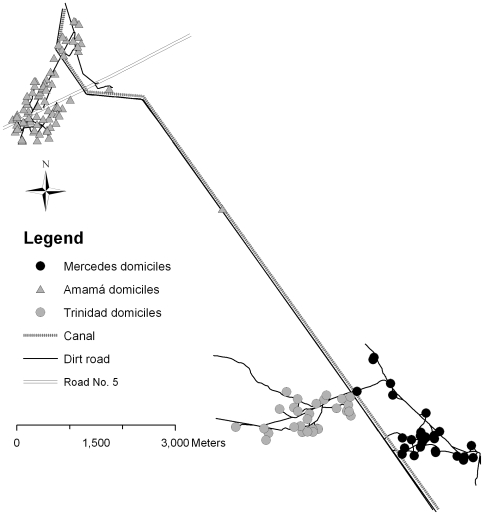
Map of the study area.

*Triatoma infestans* density data were collected in three villages in rural northwest Argentina (Amamá, Mercedes and Trinidad, 27.1°S, 63.0°W, province of Santiago del Estero, see Figure 1) after the villages were subjected to a blanket insecticide spraying in October 1992. From November 1994 to May 1999 the number of bugs was counted twice a year on all sites within a village that could potentially harbour bugs (e.g. houses, goat corrals, chicken coops, etc.). Details of the data collection are described by zu Dohna et al. [12]. Each survey noted when a site was sprayed selectively with pyrethroid insecticides. Not all sites were present at each survey since some sites were constructed or demolished during the years of observation. Most of the temporary sites were makeshift brooding sites for chickens and some were goat or pig corrals. Any newly constructed site was included in the next survey following the site's construction. The type of each site (chicken coop, goat corral, bedroom, etc., previously referred to as ‘ecotype’ [12]) was also recorded.

All sites were georeferenced and their UTM coordinates were determined (for details see [15]). Since UTM coordinates are planar projections, distances between sites were calculated using standard planar geometry.

We used all pairs of consecutive surveys (denoted survey *t* and *t*+1) to fit discrete- and continuous-time models for the probabilities of bug establishment and extinction on all sites between *t* and *t*+1. Throughout this paper, a site is called *infested at t* if one or more *T. infestans* bugs (nymphs or adults) were collected at this site at survey *t* and *uninfested at t* otherwise.

The change from uninfested to infested between two consecutive surveys is called *establishment* and the change from infested to uninfested *extinction*. The terms establishment and extinction describe observed patterns rather than population processes. A site that was observed uninfested at *t* is called a *target site* at *t*. The data show 80 instances of sites uninfested at *t* being sprayed between *t* and *t*+1. Dispersers might not emigrate from all sites infested at *t* but only from a subset which we call *source sites* at *t*.

We excluded all sites from the analysis that were never infested in any of the ten surveys but otherwise used observations from all site types including domiciliary sites. Excluding sites that were never infested in any of the ten surveys might lead to an overestimation of the average rate of bug establishment but it is unlikely to change our conclusions regarding the rate of bug establishment as a function of season and distance to source sites.

Our previous study of these data [12] did not consider the spatial location of sites and excluded domestic sites and target sites that were uninfested for only one survey. Spatial data and all target sites are included here. Including target sites that were uninfested for only one survey affects the results of neither the previous nor the current study (unpublished analyses). A total of 186 sites (30 domestic and 156 peridomestic sites) were observed during nine time intervals leading to a total number of 1396 observations (not all sites were present at all time intervals).

### Model fitting procedure

Models of increasing complexity were fitted to the data. All models had the same basic structure of predicting establishment probabilities of each target site for each time interval from *t* to *t*+1 based on the number of bugs found at other sites between *t*−1, *t* and *t*+1. Two hierarchical sets of models were evaluated in parallel, one that increments time discretely from *t* to *t*+1 and another that treats time as continuous. For both sets of models, the analysis was divided into two steps. In the first step, probabilities of establishment and extinction were analyzed only for sites that were not sprayed between *t* and *t*+1. In this step, the best model was selected from a range of alternative models which used different definitions of source sites and different patterns of seasonality in detection probability, dispersal intensity or dispersal distance. The best model selected in this procedure was used in a second step to estimate more extensive models for all target sites (sprayed and unsprayed). These more extensive models included additional effects of insecticide spraying between *t* and *t*+1 or prior to *t* and infestation prior to *t* on bug establishment between *t* and *t*+1.

### Discrete-time models

In the discrete-time models, any site can make only one transition (extinction or bug establishment) between two consecutive surveys. Discrete-time models require assumptions about the order of extinction and establishment events. If extinction happens before establishment, the probability of an observed extinction equals the probability of extinction times the probability of no re-establishment within the same time interval. If extinction happens after establishment, the probability for an observed extinction does not depend on the establishment probability. Since any assumed order is artificial we decided for mathematical convenience to make the extinction probability of a site independent of the number of bugs found on other sites. Continuous-time models (see below) are necessary to properly account for extinction and establishment within a time interval.

The probability that a site experiences bug establishment is assumed to depend on the number of bugs found on other sites. The number of dispersers that establish successfully at site *i* between surveys *t* and *t*+1 is assumed to follow a Poisson distribution whose parameter *λ_i_*(**x** *_t_*) is a function of the vector **x** *_t_* of the number of bugs found on source sites at survey *t*. The probability *p_it_* for successful bug establishment between *t* and *t*+1 on site *i* is therefore given by 1−exp(−*λ_i_*(**x** *_t_*)). The function *λ_i_*(**x** *_t_*) depends on *r_ij_*, the distance between site *i* and *j* and *x_jt_*, the bug density on site *j* at survey *t* according to
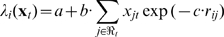
(1)Here *ℜ_t_* denotes the set of source sites at survey *t* and *a*, *b* and *c* are parameters to be estimated. The parameter *a* describes the rate of establishment not accounted for by bugs reported on other sites in the village; such establishments could come from outside the village, undetected sources within the village or apparent establishments due to erroneous failure to detect bugs at a site at time *t* (i.e. false negative at time *t*). The parameter *b* determines how strongly bugs found at *t* contribute to bug establishment on other sites between *t* and *t*+1. The parameter *c* describes how this contribution drops with distance between a source site and a target site. The estimated establishment function is similar to the approach used by Levy et al. [16], except that in our model the establishment rate itself is proportional to the number of bugs found at source sites whereas in their model it is the logarithm of the establishment rate.

The parameters *a*, *b* and *c* were estimated by maximising the log-likelihood function
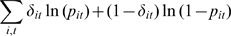
(2)where *δ_it_* equals unity if site *i* experienced bug establishment between *t* and *t*+1 and zero otherwise. The log-likelihood function was maximized via Fisher-scoring and the Newton-Raphson method [17]. Extinction probabilities (*p_e_* for unsprayed sites and *p_es_* for sprayed sites) were fitted by dividing the number of sites that went extinct between *t* and *t*+1 by the number of sites that had bugs present at survey *t* and existed at survey *t*+1. The combined log-likelihood function for extinction and establishment was the sum of equation (2) applied to extinction and establishment events.

Previous non-spatial analysis of these data [12] indicated seasonality in the slope of observed establishment events as function of the number of bugs on source sites. According to the model given by equation (1) this seasonality could be caused by seasonality in *b*, *c* or both parameters. Furthermore this seasonality could also be caused by seasonal variation in bug detection. Seasonality in *b* or *c* was estimated in the model by allowing, for example, one value of *b* for the time interval from November to May and a possibly different value of *b* for May to November; and similarly for *c*. Seasonality in bug detection was modelled by introducing a probability *p_d_* that infestation is detected during surveys in May. Lower temperatures in May could lead to decreased detection of bugs in May. Hence, the detection probability *p_d_* was introduced for May surveys whereas November surveys were assumed to always detect infestation on a site given bugs were present. The true detection probability for November surveys is most likely significantly less than 100%. However, this analysis is less concerned with estimating the true detection probability but rather the effect of a relative decrease of detection in May. Allowing for undetected infestation introduces an unobserved variable. Integrations over unobserved variables can be computationally expensive (e.g. [18]), however in our case we simply had to sum over the two possible infestation states of sites that were observed uninfested in May (for details see Appendix S1).

If there is no spatial association between bug establishment and source sites, the parameter *c* equals zero. We therefore compared the bias-corrected Akaike information criterion (AIC_c_) of eight different model structures that arose from combining the four alternatives of seasonality (seasonality in (I) *c*, (II) *b*, (III) *c* and *b*, or (IV) *p_d_*) with the two alternatives for spatial structure ((A) *c*>0 and (B) *c* = 0, i.e. lower *c* = 0 for models with seasonality in *c*).

Another model component is *ℜ_t_*, the set of source sites at survey *t*. A non-spatial discrete-time model for the same data [12] suggested that among all sites infested at *t*, only sites which were also infested at *t*−1 and *t*+1contributed to bug establishment on other sites between *t* and *t*+1. The eight model structures above were therefore combined with the following three alternative ways to estimate the source of dispersers for the time interval from *t* to *t*+1: (i) *ℜ_t_* is given by all sites infested at *t* and *x_jt_* by the total number of bugs per site at time *t*, (ii) *ℜ_t_* is given by all sites that were infested at *t*−1, *t* and *t*+1 and *x_jt_* by the total number of bugs at site *j* at time *t*, (iii) *ℜ_t_* is given by all sites infested at *t* and *x_jt_* by the sum of the number of adults and fifth instar nymphs at site *j* at time *t*.

### Effects of insecticide spraying in discrete-time models

The best model (i.e. the one producing the lowest AIC_c_ value) among the 24 models that were fitted to unsprayed target sites was used as a basis to fit models that incorporated additional effects of insecticide spraying and infestation history on bug establishment. Two alternative models for the effect of insecticide spraying on bug establishment were fitted. According to the first model, insecticide spraying between *t* and *t*+1 reduces bug establishment only within the same time interval. This model multiplies the establishment function *λ_i_*(**x** *_t_,t*) of equation (1) by the factor *ρ* for each site that was sprayed between *t* and *t*+1, i.e. *λ′_i_*(**x** *_t_,t*) = *ρλ_i_*(**x** *_t_,t*). Here the prime indicates the modified establishment function, not a derivative.

The second model includes two spraying parameters *α* and *β* to allow for short- and long-term effects of insecticide spraying. This model modified the establishment function of equation (1) to *λ′_i_*(**x** *_t_,t*) = *λ_i_*(**x** *_t_,t*) (1−exp[−*α*−*β* (*t*−*t*_0*i*_)]) where *t*_0*i*_ denotes the beginning of the last time interval during which site *i* was sprayed. Hence when site *i* was sprayed between *t* and *t*+1, *t*−*t*_0*i*_ = 0. The model for short-term effects is a special case of this model with *β* = 0 and *ρ* = (1−exp[−*α*]). The parameter *α* describes by how much bug establishment is reduced during *t* and *t*+1 by spraying during the same time period. The parameter *β* describes how fast the effect of spraying on bug establishment decays over time.

The reduction of establishment between *t* and *t*+1 due to spraying in the same time interval (as described by parameter *α*) can be due to two effects – instantaneous extinction of sites that experienced establishment before they were sprayed and reduced establishment in the time from spraying to *t*+1. Teasing apart these two effects necessitated a continuous-time model as described below.

The most parameter-rich discrete-time model added to the effect of spraying an effect of time since last infestation on bug establishment. Bugs at a site could influence the site's post-extinction probability of re-establishment if they left eggs that hatch later, bug attractants, or any other marking substance that affects other bugs' behaviour. For example, bug feces have some substances used by bugs to mark a refuge's entrance. Many of these effects might increase establishment rates after previous infestation, however, our previous analysis of the same data [12] did not detect any positive associations between the subsequent establishment events on the same site. We therefore tested in this study for the presence of a negative effect of time since last infestation on bug establishment. This effect was assumed to decay over time with decay rate *γ*. Hence, in the most complex model the establishment function of equation (1) was modified to *λ′_i_*(**x** *_t_,t*) = *λ_i_*(**x** *_t_,t*) (1−exp[−*α*−*β*(*t*−*t*_0*i*_)]) (1−exp[−*γ* (*t*−*t*_1*i*_)]) where *t*_0*i*_ is as before and *t*_1*i*_ denotes the last survey at which site *i* was infested. Recall that the site must be uninfested at time *t*, so *t*_1*i*_ must be *t*−1 or earlier.

Any change in marginal establishment rate after the last infestation could be an artifact caused by an underlying trend in establishment rate since all but one site started uninfested in the first survey and the time since last infestation increased for most sites during the study. We therefore tested whether the reduced bug establishment after previous infestation can still be observed when the time since last infestation is ignored. For both villages, we counted how often establishment events were observed on sites that had already experienced establishment in a previous time step and compared this number to numbers obtained through Monte Carlo simulations. In our simulations, we selected for each survey *t* from the set of target sites at *t*, randomly and without replacement, a number of sites equal to the number of establishment events observed between *t* and *t*+1. For each sampled establishment event, we counted how often this site experienced establishment in previous surveys according to the observed data. To exclude effects of spraying on the probability of re-establishment after infestation, we restricted this analysis to target sites that were last sprayed during the blanket insecticide campaign before the surveillance period. In the simulations establishment events were either sampled with equal probability for all target sites (unweighted sampling) or according to establishment probabilities estimated by the best model without the *γ*-parameter (weighted sampling).

### Continuous-time models

Continuous-time equivalents of the discrete-time models were also fitted. A continuous-time model can estimate within-season changes of establishment dynamics and time-dependent effects of insecticide spraying, but requires explicit assumptions about how the sources of dispersers change between *t* and *t*+1.

**Figure 2:**
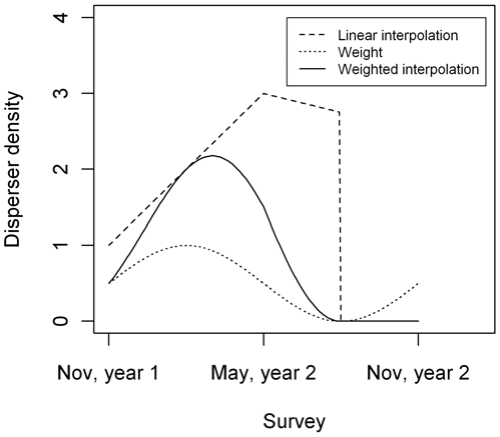
Reconstructed disperser density within a season of a hypothetical source site. This site had one bug in November of year 1, three bugs in May of year 2 and was sprayed in August of year 2. The slope between May of year 2 and spraying is the average slope of all unsprayed sites. The weighted interpolation is the product of the linear interpolation and weight function (see text) whose shift parameter equals 1 in this example – corresponding to peak dispersal end of January.

The contribution of each source site to bug establishment between *t* and *t*+1 was estimated by linear interpolation between the number of bugs found at the source site at *t* and the number of bugs found at *t*+1 (Figure 2). The bug density of sites that were sprayed at time Δ between *t* and *t*+1 was assumed to follow the average change of bug density on unsprayed sites from *t* to Δ and to equal zero from Δ to *t*+1 (Figure 2). The same source site definitions (i) and (iii) as used for discrete-time models were tested for the continuous-time models. Source site definition (ii) of discrete-time models was replaced for the continuous-time models by definition (ii′). According to definition (ii′) *ℜ_t_* is given by all sites that were infested at *t*−1 and *t* since in the continuous-time models extinction of source sites between *t* and *t*+1 is accounted for by the interpolation method described above. Instead of estimating different values of *b* for different seasons, seasonality in dispersal intensity is described for the continuous-time model by a sinusoidal weight function *w*(*τ*) with period two (i.e. two seasons for one year) and a shift parameter that determines the location of the peak dispersal season (see Appendix S1). Figure 2 illustrates the two methods to reconstruct within-season dispersal.

In the continuous-time model, all instantaneous effects of establishment at one site on establishment on other sites were ignored. This assumption was based on results from previous analyses [12] which found evidence for a time lag between bug establishment on a site and this site's contribution to bug establishment on other sites. The same term as in the discrete-time models was used to describe effects a site has on its own establishment rate after extinction (the term assumes negative effects and the parameter *γ* describes the rate at which such effects disappear). We did not allow a site to influence its own establishment rate within the same time interval through the establishment function *λ_i_*(**x** *_t_,t*) because *λ_i_*(**x** *_t_,t*) is meant to describe dispersal processes, which are very different from the processes which influence the site's post-extinction probability of re-establishment. Ignoring positive effects of previous infestation on subsequent establishment is likely to inflate parameter *a* in the establishment function. Parameter *a* estimates the rate of establishment that cannot be attributed to other source sites. On the other hand, subsuming positive effects of previous infestation on subsequent establishment in the establishment function influences parameter *c*, since these effects are treated as dispersal between sites with zero distance. Since our analysis is more concerned with estimating distance effects than the intercept parameter *a* we fitted a model in which no target site can be its own source site.

The formalism of continuous-time Markov processes [19] was used to derive transition probabilities for observations at surveys (for details, see Appendix S1). The maximum likelihood parameters were estimated using Fisher-scoring [20].

### Effects of insecticide spraying in continuous-time models

The continuous-time models allowed a more detailed estimation of the effects of insecticide spraying. Insecticide spraying can affect a local bug population by (i) causing instantaneous extinction (which occurs with probability *p_I_*) and (ii) reducing subsequent establishment on this site over the time period in which residual insecticide effects persist. The details of estimating the instantaneous extinction probability are explained in the Appendix S1. To account for the reduction of subsequent *λ* is multiplied by (1−exp[−*β* (*τ*−*τ*_0*i*_)]). As in the discrete-time model, *τ*_0*i*_ denotes the time since last spraying.

### Deviations from model assumptions

For the best fitting discrete-time model and the best fitting continuous-time model, the model assumptions were tested several ways. Model residuals were tested for overdispersion. ANOVAs of Pearson residuals were used to test whether the probabilities that a site would experience establishment differed by site type and whether sites that did not exist at *t* (i.e., newly created sites) were less likely to experience establishment between *t* and *t*+1 than sites that did exist at *t*. The latter could indicate that some sites that changed from uninfested at *t* to infested at *t*+1 might have experienced bug establishment earlier that went undetected, since changes from uninfested at *t* to infested at *t*+1 due to erroneous failure to detect establishment at *t* can occur only on sites that existed at *t*. To determine how the parameters of the best model depend on deviations of all other parameters, we successively perturbed each parameter to 10% above and below its maximum likelihood estimate and re-estimated all other parameters for each parameter perturbation.

## Results

**Figure 3:**
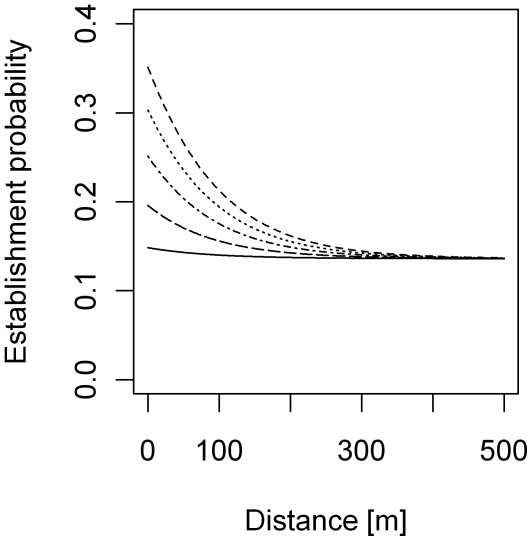
Establishment probability vs. distance from source site. The lines were drawn using maximum likelihood parameters for November–May. Different lines correspond to 1 (solid line), 5 (dashed line with short spaces), 10 (dashed-dotted line), 15 (dotted line) and 20 (dashed line with long spaces) bugs found at the source site. The establishment probabilities do not depend on distance for time period May–November and equal 0.15, 0.20, 0.25, 0.30 and 0.35 for 1, 5, 10, 15, and 20 bugs at the source site, respectively.

Regardless of model type (discrete- vs. continuous-time) or source-site definition, all the best models included seasonality in dispersal distance and at least one positive estimate of parameter *c* (Table 1). The model based on seasonality of detection (parameter *p_d_*, Table 1) performed worst for all model types. In all the best models, bug establishment was more concentrated around source sites (i.e. they all had a higher *c*-value) in the period from November to May than May to November (Figure 3). The lower *c*-values for the period from May to November also indicate higher establishment rates on the village level during the same time period (i.e. the curves in Figure 3 averaged over all distances are lower than the constant establishment rates for May–November). Whether the best model included seasonal variation in dispersal intensity (parameter *b*) depended on whether time was treated as continuous or discrete and the definition of source sites. When sprayed target sites were excluded, the overall best model was the discrete-time model with source-site definition (ii), counting all bugs from sites that were infested at *t*−1, *t* and *t*+1 (Table 1). All six models with discrete time and source-site definition (ii) were far superior, according to the bias-corrected AIC_c_-values, to any of the 42 other models.

**Table 1:** Bias-corrected AIC_c_-values of models that consider only data from unsprayed target sites and make different assumptions about seasonality of dispersal intensity parameter *b* and distance parameter *c*.

Time	Source	(I) seasonality in *c* only		(II) seasonality in *b* only		(III) seasonality in *b* and *c*		(IV) seasonality in *p_d_*	
		(A) both *c*>0	(B) lower *c* = 0	(A) *c*>0	(B) *c* = 0	(A) both *c*>0	(B) lower *c* = 0	(A) *c*>0	(B) *c* = 0
discrete	(i) all bugs from sites infested at *t*	929.6	938.5	933.4	936.6	926.4*	927.7	944.8	938.5
	(ii) all bugs from sites infested at *t*−1and *t*, and *t*+1	917.6	916.5*	920.4	919.9	919.3	918.4	939.7	935.5
	(iii) adults and 5^th^ instar nymphs from sites infested at *t*	927.8	934.3	931.0	938.3	925.8*	930.6	947.2	937.7
continuous	(i) all bugs from sites infested at *t*	934.4	936.1	939.4	938.3	933.6*	937.1	945.4	951.2
	(ii′) all bugs from sites infested at *t*−1 and *t*	926.7*	930.9	929.3	931.9	928.4	930.0	945.8	940.7
	(iii) adults and 5^th^ instar nymphs from sites infested at *t*	938.1*	939.4	938.5	939.8	938.5	938.9	949.9	957.9

*: The model with the lowest AIC_c_-value in each row.

**Figure 4:**
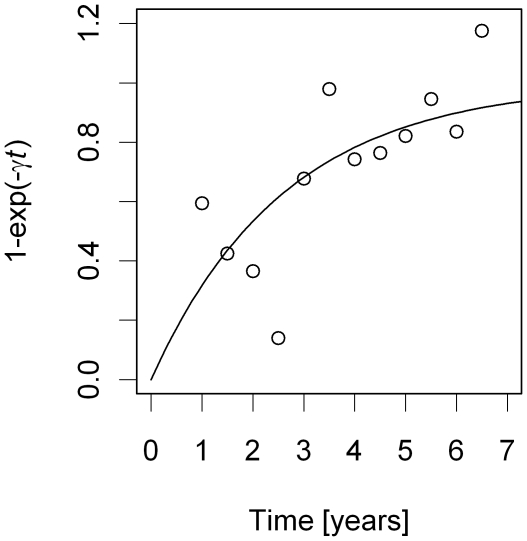
Effect of time since last infestation on marginal establishment rate *λ*. The dots indicate, for the best model without the γ-parameter, the ratio of observed over expected number of establishment events per site pooled over all sites with the same time since last infestation. The values were multiplied by the ratio of the mean of the observed ratios over the mean of the curve such that the observed ratios and predicted curve give the same mean.

**Figure 5:**
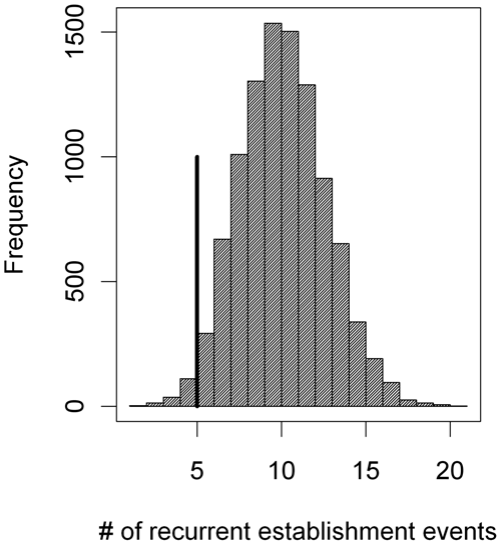
Simulated and observed number of establishment events on previously infested sites. Grey bars denote observed numbers and the black bar shows the simulated number of establishment events. All numbers are for Amamá and Mercedes-Trinidad combined.

**Figure 6:**
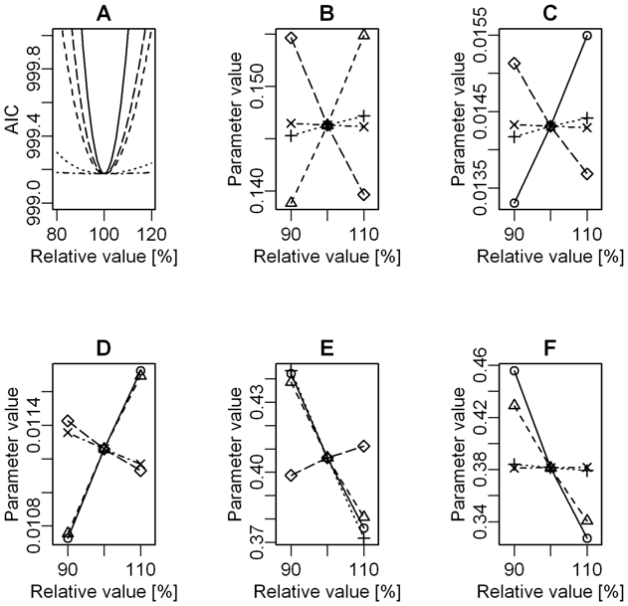
Sensitivity analysis. AIC of best model as function of deviation from the maximum likelihood estimates (A). The *x*-axis shows the parameter deviation as percentage (maximum likelihood estimate = 100%, dashed-dotted line denotes *ρ*, dotted line denotes *c*_1_, short dashed line denotes *b*, long dashed line denotes *γ* and solid line *a*). Panels B–F show how the best estimate of a parameter (*y*-axis) varies as function of percent deviations of all other parameters (*x*-axis) for *a* (B), *b* (C), *c*_1_ (D), *ρ* (E) and *γ* (F). Line symbols are the same as in panel A.

**Figure 7:**
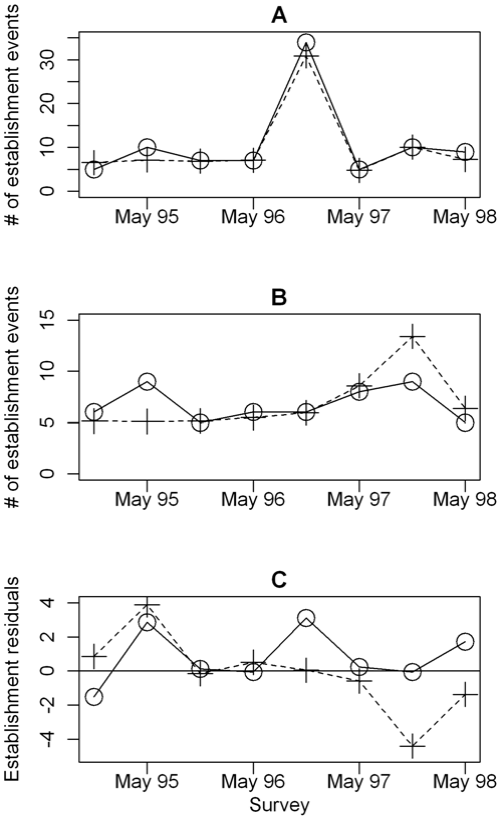
Number of establishment events between *t* and *t*+1. Observed (solid line with circles) and predicted number of establishment events by best model (dashed line with crosses) for Amamá (A) and Mercedes-Trinidad (B) plotted against survey date at *t*. Panel C shows the residuals from A (solid line with circles) and B (dashed line with crosses).

When the effect of insecticide spraying on target sites was included, the discrepancy in AIC_c_ between discrete- and continuous-time models decreased, but discrete-time models still outperformed continuous-time models (Table 2). According to the best discrete-time model, spraying decreases the colonization probability by 60% within the same season but has no long-term effect (Table 3). The best model also included an effect of infestation history on establishment probability (Figure 4). The rate of establishment after infestation returned very slowly back to previous values within seven years (Figure 4). This effect occurred after the effect of insecticide spraying was accounted for. The same negative effect of previous infestation on subsequent establishment rates emerged from the Monte-Carlo simulations which predicted, for both weighted and unweighted sampling, a number of establishments on previously infested sites higher than was observed in the data (P-value = 0.02, Figure 5). The AIC values for the best model show a minimum around the best estimates for all parameters; however this minimum is very flat for *ρ* (Figure 6A). Changing *ρ*, on the other hand, has very little influence on the best estimates of the other parameters (Figure 6 B–F). The residual deviation between the observed and predicted number of establishment events per survey is correlated between the village of Amamá and the combined villages of Mercedes and Trinidad (Figure 7).

**Table 2:** Bias-corrected AIC_c_-values of models that consider sprayed and unsprayed target sites and fit different numbers of parameters.

Model time and source definition	Estimated effects of spraying on bug establishment	Effects of previous infestation on bug establishment (*γ*) estimated	
		yes	no
discrete (ii)	Short term effect (*ρ*) only	999.2	1004.6
	Short and long term effects (*α* and *β*)	999.9	1005.2
continuous (ii′)	Effect on establishment (*β*) only (*p_I_* = 1)	1002.0	1006.3
	Effect on establishment (*β*) and probability of instantaneous extinction (*p_I_*)	1004.0	1008.3

In every case, estimating the effects of previous infestation on bug establishment gave the lower AIC_c_-value, indicating the better fit.

**Table 3:** List of all parameters. Maximum likelihood values are provided for parameters that were part of the best model (discrete-time model with all bugs from sites infested at t−1, t and t+1 counted as source). For details on parameters see equation (1) and text.

Symbol	Description	Value in best model
*a*	Intercept for establishment function (determines the rate of establishment not attributable to other source sites)	0.15
*b*	Slope of establishment function (determines the increase in the rate of establishment as function of bugs found on other source sites)	0.014
*c*_1_	Distance decay parameter for intervals from November–May [m^−1^]	0.011
*c*_2_	Distance decay parameter for intervals from May–November [m^−1^]	0 (not fitted)
*p_e_*	Extinction probability for unsprayed sites in discrete-time model	0.5
*p_es_*	Extinction probability for sprayed sites in discrete-time model	0.9
*p_d_*	Probability to detect infestation at a site in May surveys given the site is infested	NA
*p_I_*	Probability of instantaneous extinction due to spraying in continuous-time model	NA
*μ*	Extinction rate in continuous-time model	NA
*ρ*	Proportionality factor for establishment on sites sprayed within the same time interval	0.4
*γ*	Factor for increase of establishment rate after last infestation [year^−1^]	0.38
*α*	Factor for instantaneous effect of spraying on establishment rate	NA
*β*	Decay rate for on establishment rate [year^−1^]	NA

Neither of the models shown in Table 2 showed significant overdispersion. The establishment probability between *t* and *t*+1 did not differ significantly among sites that did or did not exist at *t* (*P* = 0.21). Pearson residuals did not vary significantly with site type (ANOVA, *P* = 0.79) hence establishment rates did not differ significantly between domestic and peridomestic sites.

## Discussion

Discrete- and continuous-time Markov models for site-level transitions from uninfested to infested and vice versa were fitted to spatiotemporal population data of *T. infestans*, the vector of Chagas disease. Both sets of models showed a strong seasonality in bug establishment on new sites by *T. infestans* coincident with seasonality in dispersal distance.

While both sets of models produced the same general result, discrete-time models produced lower AIC_c_ values, and therefore fitted the data better than continuous-time models. When only unsprayed target sites were considered (Table 1), the superiority of discrete-time models was much more pronounced than when unsprayed and sprayed target sites were considered (Table 2). Continuous-time models require specific assumptions of how the number of dispersers emanating from a site changes over time between two consecutive surveys. The poorer performance of continuous-time models shows that these assumptions are too restrictive for our data. Continuous-time models, however, allow decomposing the effects of spraying on target sites into instantaneous extinction and reduction of subsequent establishment, reducing the advantage of discrete-time models when sprayed target sites are included in the analysis (Table 2).

The model comparison produced interesting and some surprising results that could be highly relevant for vector control. The analysis presented here confirmed previous results showing [12] that sites that were not infested in a previous survey did not contribute to bug establishment on other sites. This suggests that there is a time lag between bug establishment on a site and dispersal from this site. In contrast to the earlier non-spatial analysis the more detailed analysis here shows how properties of individual target sites such as their distance from source sites, their infestation history and spraying history contribute to their rate of bug establishment.

To optimize the timing of vector control, it is crucial to understand the seasonality of bug dispersal intensity and distance (and not only the duration of tethered flight). Our analysis showed higher rates of bug establishment from May to November than from November to May, consistent with our previous results [12]. We found no evidence that this pattern was caused by a lower detection probability during the May surveys. In contrast to these results, Gurevitz et al. [13] found a high tendency of *T. infestans* to initiate dispersal flight in experimental huts from late February to April, and evidence from light traps [14] as well as bug collections by householders [21] suggest that more *T. infestans* disperse from November to May than from May to November. The dependence of flight initiation on temperature [13] and nutritional status [22] when compared to seasonality in temperature and host availability makes initiation of dispersal during mid fall-winter months (May to September) very unlikely. More information is required to resolve the conflicting evidence regarding the duration and detailed time structure of the dispersal season.

The higher rates of establishment between May and November suggested by our study coincide with longer dispersal distances during the same time period (Figure 3). According to the best model, between May and November no decrease of the establishment probability with distance was detected within a village (maximum distance ∼2 km). Our results suggest that between May and November a considerable proportion of bugs might disperse further than 1.5 km. Observations of dispersal distance are very sparse. *T. infestans* has been shown to cover a distance of 1.5 km in an open field [23] and can sustain tethered flight for over 2.4 km [24]. Here we demonstrate that the previously shown potential for long range dispersal of *T. infestans* is reflected in patterns of bug establishment for the season from May to November. For the season from November to May, the decrease in establishment probability with distance from source sites is flatter than a previous estimate for *T. infestans* [16]. The discrepancy between the previous study and our own can be explained by the difference in the proportion of early instar nymphs among bugs found on target sites. Early instar nymphs which disperse over smaller distances made up a much higher proportion in the previous study than in ours, possibly because of shorter distance between sites in the previous study (e.g. the proportion of 1^st^ instar nymphs on target site was less than 5% in our study and more than 50% in theirs).

Two mechanisms could link larger dispersal distances with a higher number of bug establishments. When the number of target sites is limiting, short dispersal distances could lead to multiple bug establishments on the same site, thereby limiting the total number of sites experiencing bug establishment. Since establishment probabilities are not close to unity in the vicinity of high-density source sites (Figure 3, see also [3]), target sites were not limiting (especially during 1992–1996 and after 1997) and hence the link between dispersal distance and overall number of establishments must be due to a different mechanism.

An alternative mechanism linking dispersal distance and intensity is mortality during bug dispersal (e.g. [25]). Even without any seasonality in dispersal behavior, seasonality in mortality alone could create the observed seasonality in bug establishment, where the high-mortality season has fewer establishment events and a shorter average dispersal distance while the low-mortality season has more establishment events and a larger average dispersal distance. During the low-mortality season fewer dispersers die before they reach a destination leading to a larger number of successful establishment events. Also, during that season each disperser moves on average for a longer time period leading to longer average dispersal distance than in the high-mortality season. The observed pattern in establishment seasonality can therefore be a product of seasonality in flight initiation, flight activity, mortality and possibly other components of *T. infestans* ecology. For all these factors, very few field data are available.

The following hypothetical scenario could reconcile previous results with the patterns found in this study: Adults start dispersing in late summer (March–April) but the majority might not settle on new sites by May and would therefore be difficult to detect in May surveys. Yet population sizes from May surveys might be a good approximation of population sizes in March and therefore proportional to the number of dispersers. Bugs settling in new sites from May to November experienced a longer dispersal phase and settle therefore further away from their source sites. In agreement with this scenario, Gorla and Schofield [26] observed that, among cohorts of females emerging in closed experimental huts under natural climatic conditions in northwest Argentina, the proportion of females surviving the first three months of adulthood was 70–100% for females emerging in April (i.e., from fall to winter) and 10–50% for females emerging in October (from spring to summer). Although closed experimental huts differ from field sites regarding death risks and flight dispersal, this mortality pattern is probably applicable to the field if it is due only to temperature variations.

While the proposed scenario is a parsimonious explanation of the results of this and previous studies, it would imply that the majority of dispersing females settle on new sites 2–3 months after they initiated dispersal. Although theoretically possible this would be very unusual. Another possibility to reconcile the results presented here with previous results is a pulsed dispersal phase in September–October that previous light trapping experiments did not detect.

Both scenarios, a pulsed dispersal or a dispersal phase during which bugs are neither found on source or target sites, deviate from the mechanisms assumed by the continuous-time models fitted here. Since continuous-time models are more explicit about the underlying dispersal mechanisms, a continuous-time model that captures the proper dispersal mechanism should fit better than a discrete-time model. We therefore propose that future work should extend continuous-time models to account for patterns of bug dispersal obtained in other experiments. Another important step towards more realism would be to include within-site population dynamics. The models we have fitted assume that extinction risk is independent of local population size and treat all establishment events the same, regardless of how many bugs were found after establishment. Fitting models that utilize information of local population size could allow a better understanding of the dispersal process. However, more complex models have to be based on a better understanding of the underlying processes, which ultimately comes from better data.

There are no direct observations of dispersal behavior and mortality of free-living *T. infestans* individuals. Our results are based on indirect inferences drawn from patterns of bug occurrence in the field. Other studies have addressed components of the dispersal process by observing flight initiation in experimental settings or counting the number of bugs trapped in UV light. While each of these studies contributes important pieces, our analysis suggests that more detailed observations of bug dispersal are necessary to complete our understanding of the seasonality in dispersal distance and intensity. To determine the seasonality and length of dispersal phases for individual bugs would require a long-term mark-release study of bugs in experimental huts within a large scale enclosure. This experiment would not reflect accurately dispersal distances in the field but would permit comparisons between seasons of rates of dispersal initiation and mortality. The experimental huts would have to be far enough apart that movement between them would require an active act of dispersal (e.g. 20–50 m apart).

Our analysis also aimed at understanding the temporal effects of insecticide spraying. According to the best model, spraying reduces bug establishment within a six-month period but not beyond (Table 2). This estimate is consistent with the quick decay of pyrethroid insecticides exposed to the sun [27].

A surprising result of our analysis is that, after controlling for the effect of spraying, there is a long-term trend of increasing marginal rate of establishment with increasing time since last infestation (Figure 4). However, the reverse may be true within the first 2.5 years since the last infestation because the first four points in Figure 4, which encompass a total of 257 observations, display a short-term downward trend. We have done no test to determine whether this downward trend could be due to random fluctuations. At face value, in the short run the establishment rate seems higher on sites that were infested more recently, which is consistent with observations that *T. infestans* feces can attract conspecifics [28],[29],[30] and with experimental evidence that recently infested sites experience higher establishment rates than recently uninfested sites [27].

The long-term increase of establishment rates after previous infestation that is also supported by the results of our Monte-Carlo simulations (Figure 5) is more difficult to explain since bugs are usually attracted by signs of conspecifics [28],[29],[30] (but for an exception see [31]). Since the change of establishment rate after extinction is highly relevant for designing optimal insecticide spraying schedules, it is important to conduct further experiments to understand the biological mechanisms underlying the observed pattern of slowly increasing marginal establishment rate after previous infestation.

Even though the best model can explain the main patterns of bug establishment (Figure 7 A and B), the residuals systematically vary similarly over time for Amamá and combined Mercedes and Trinidad (Figure 7 C). Since all villages were subjected to blanket spraying at the same time, these correlated residuals could indicate long-term effects due to internal dynamics. Alternatively, the residuals could reflect some large-scale external forcing (not yet identified) that acted similarly on both villages, such as clearing of surrounding forest or weather. Explaining this pattern of residuals shared between villages is likely to provide a deeper understanding of *T. infestans* dynamics.

## Supporting Information

Appendix S1 Details of statistical analysis. Details of the fitting procedure for models with detection seasonality and continuous time models.
(0.07 MB DOC)
