# Supplementary material for: Spatial Re-Establishment Dynamics of Local Populations of Vectors of Chagas Disease
Source: PLoS Negl Trop Dis. 2009 Jul 28;3(7):e490. doi: 10.1371/journal.pntd.0000490 (PMC2709728; doi:10.1371/journal.pntd.0000490)
Supplement: Appendix S1 — Details of statistical analysis. Details of the fitting procedure for models with detection seasonality and continuous time models. (0.07 MB DOC) [file pntd.0000490.s001.doc]

Appendix S1:

*A note on definitions*

Our previous study of these data [1] defined the combination of arrival and persistence to the next survey as establishment. In the present paper, the change from uninfested to infested between two consecutive surveys is called establishment. Our previous definition posited underlying mechanisms. The present definitions refer to observable patterns. Both definitions mean the same thing but the latter is more readable.

In [1], we defined a target site at *t* to be a site that was observed uninfested at *t*‑1 and *t*. In the present paper, a site that was observed uninfested at *t* is called a target site at *t*. Whether or not target sites that were uninfested for only one survey are included affects the results of neither the previous nor the current study (unpublished analyses).

*Model with detection seasonality*

The model below allows sites to be infested but undetected during May surveys. The biological justification for this modification is that lower temperatures in May could lead to decreased detection of bugs in May, which could explain the higher rate of observed bug establishment between May and November. This model requires a distinction between observed and true states of a site. We will use the following notation for the four possible states of a site:

*u_t_* : site is observed uninfested at survey *t*

*i_t_* : site is observed infested at survey *t* (and therefore must be truly infested)

*u’_t_* : site is truly uninfested at survey *t*

*i’_t_* : site is truly infested at survey *t*

For the time intervals from November to May, the transition matrix for the observed states becomes:

where *p_d_* denotes the probability to detect infestation on a site given it is there. *P*(*s’_t_*_+1_|*r’_t_*) denotes the transition probabilities for the true states, i.e. the probability that a site is in state *s’* at time *t*+1 given that it was in state *r’* at time *t*. The transition probabilities for the true states are the same as in the text. The transition matrix for the observed states for the time intervals from May to November is given by:

The probabilities of the true state given the observed state can be calculated using Bayes’ theorem:

For sites that existed at *t* and were not sprayed between *t*-1 and *t*, the probability of being truly infected in May, *P*(*i’_t_*), equals *P*(*i’_t_*|*u’_t_*_-1_) if the site was observed uninfested in the preceding November and *P*(*i’_t_*|*i’_t_*_-1_) otherwise. For sites that did not exist at *t* or were sprayed between *t*-1 and *t, P*(*i’_t_*) was set to zero.

*Continuous-time model*

According to the continuous model, transition probabilities for site *i* between infested and uninfested between survey *t* and *t*+1 resulted from a continuous-time Markov process with transition intensity matrix **Q***_it_*(*τ*)

$\mathbf{Q}_{it}(\tau)=\left(\begin{matrix} -\lambda_{i}(\mathbf{x}_{t}(\tau),\tau) & \lambda_{i}(\mathbf{x}_{t}(\tau),\tau) \\ \mu& -\mu\end{matrix}\right)$ (3)

where *μ* denotes the extinction rate (i.e. the rate of transition from infested to uninfested), *τ* is the fraction of the time between *t* and *t*+1 (at *t*, *τ* =0 and at *t*+1, *τ* = 1). The bug establishment rate is

$\lambda_{i}(\mathbf{x}_{t}(\tau),\tau)=a+w(\tau)\cdot b\cdot\sum_{j\in\Re_{t}} x_{jt}(\tau)\exp(-c\cdot r_{ij})$ (4)

Seasonal variation of *b* was determined by estimating the shift-parameter *d* of a sinusoidal weight function *w*(*τ*)

$w(\tau)=\frac{1+\sin(\pi(\tau+d+step(t)))}{2}$ (5)

where *step*(*t*) indicates a step function which equals unity if *t* is odd (time intervals from November to May) and zero otherwise. The shift parameter *d* indicates the season of peak dispersal (e.g. peak dispersal in end of January would correspond to *d* = 1).

The transition probability matrix for each site was given by

$$\mathbf{P}_{it}=\exp(\int_{0}^{1} \mathbf{Q}_{it}(\tau)d\tau)$$

The integral of the *Q* matrix is obtained by integrating its elements. The integral of the establishment function from time zero to one equals for the most complex model

$$a+b\cdot\sum_{j\in\Re_{t}} x_{0jt}\exp(-c\cdot r_{ij})\int_{0}^{1} \exp\left[-\beta\cdot(\tau_{0j}+\tau)\right]\cdot w(\tau)d\tau+\sum_{j\in\Re_{t}} x_{1jt}\exp(-c\cdot r_{ij})\int_{0}^{1} \exp\left[-\beta\cdot(\tau_{0j}+\tau)\right]\cdot\tau\cdot w(\tau)d\tau$$

where *x*_0_*_jt_* and *x*_1_*_jt_* indicate the slope and intercept of the linear interpolation of the bug density at site *j* (see Figure 1). Denoting the integrals of *μ* and *λ* by *M* and *Λ* respectively the matrix for the transition probabilities becomes [2]

$$\mathbf{P}_{it}=\frac{e^{-(M+\Lambda)}}{\Lambda+M}\left[\begin{matrix} e^{M+\Lambda}M+\Lambda& \Lambda\cdot(e^{M+\Lambda}-1) \\ M\cdot(e^{M+\Lambda}-1) & e^{M+\Lambda}\Lambda+M \end{matrix}\right]$$

The derivatives of **P***_it_* with respect to each parameter were calculated using the symbolic calculation software *Mathematica*.

To estimate the probability of instantaneous extinction, *p_I_*, at any site that was sprayed at time *t*+Δ (0<Δ<1), the time interval between *t* and *t*+1 was divided into two intervals, from *t* to *t*+Δ and from *t*+Δ to *t*+1. Then the probability *P*(*I_t_*_+1_| *I_t_*) of a site’s infestation status at *t*+1 given its infestation status at *t* and spraying at *t*+*Δ* is given by

$$\begin{matrix} P(\left.I_{t+1}\right|I_{t})=P(\left.I_{t+1}\right|I_{t+\Delta}=1)\cdot P(\left.I_{t+\Delta}=1\right|I_{t})(1-p_{i})+ \\ +P(\left.I_{t+1}\right|I_{t+\Delta}=0)\cdot\left[P(\left.I_{t+\Delta}=1\right|I_{t})p_{i}+P(\left.I_{t+\Delta}=0\right|I_{t})\right] \end{matrix}$$

where *I_t_* =1 and *I_t_* =0 denote the events of a site being infested or uninfested at *t*, respectively. Hence, for a site that was sprayed at time *t*+Δ, the matrix **P***_it_* for the transition probabilities is given by

$$\mathbf{P}_{it}=\exp\left[\int_{0}^{\Delta} \mathbf{Q}_{it}(\tau)d\tau\right]\cdot\mathbf{A}\cdot\exp\left[\int_{\Delta}^{1} \mathbf{Q}_{it}(\tau)d\tau\right]$$

where the matrix **Q** is the same as in equation (3), except that *λ* is multiplied by (1 – exp[– *β*(*τ*–*τ*_0_*_i_*)]). As in the discrete-time model, *τ*_0_*_i_* denotes the time since last spraying. In the continuous-time model, this time is measured in continuous time. The matrix **A** describes instantaneous extinction due to spraying

$$\mathbf{A}=\left(\begin{matrix} 1 & 0 \\ p_{I} & 1-p_{I} \end{matrix}\right)$$

Incorporating the two effects of spraying requires *p_I_* and *β* as two additional parameters compared to the basic model in equation (1). The fit of this model was compared with the fit of a simpler model that assumes that spraying always leads to instantaneous extinction (i.e. *p_I_* =1).

**References**

1. Dohna HZ, Cecere MC, Gürtler RE, Kitron U, Cohen JE (2007) Re-establishment of local populations of vectors of Chagas disease after insecticide spraying. J Appl Ecol 44: 220-227.

2. Strang G (1998) Introduction to Linear Algebra. Wellesley, MA: Wellesley-Cambridge Press.
